# Microglia are crucial regulators of neuro-immunity during central nervous system tuberculosis

**DOI:** 10.3389/fncel.2015.00182

**Published:** 2015-05-18

**Authors:** Jonathan Paul Spanos, Nai-Jen Hsu, Muazzam Jacobs

**Affiliations:** ^1^Division of Immunology, Faculty of Health Sciences, Institute of Infectious Disease and Molecular Medicine, University of Cape TownCape Town, South Africa; ^2^National Health Laboratory ServiceJohannesburg, South Africa

**Keywords:** microglia, *Mycobacterium tuberculosis*, central nervous system, cytokines, chemokines, neurotoxicity, neuroprotection, tuberculosis meningitis

## Abstract

*Mycobacterium tuberculosis* (*M. tuberculosis*) infection of the central nervous system (CNS) is the most devastating manifestation of tuberculosis (TB), with both high mortality and morbidity. Although research has been fueled by the potential therapeutic target microglia offer against neurodegenerative inflammation, their part in TB infection of the CNS has not been fully evaluated nor elucidated. Yet, as both the preferential targets of *M. tuberculosis* and the immune-effector cells of the CNS, microglia are likely to be key determinants of disease severity and clinical outcomes. Following pathogen recognition, bacilli are internalized and capable of replicating within microglia. Cellular activation ensues, utilizing signaling molecules that may be neurotoxic. Central to initiating, orchestrating and modulating the tuberculous immune response is microglial secretion of cytokines and chemokines. However, the neurological environment is unique in that inflammatory signals, which appear to be damaging in the periphery, could be beneficial by governing neuronal survival, regeneration and differentiation. Furthermore, microglia are important in the recruitment of peripheral immune cells and central to defining the pro-inflammatory milieu of which neurotoxicity may result from many of the participating local or recruited cell types. Microglia are capable of both presenting antigen to infiltrating CD4^+^ T-lymphocytes and inducing their differentiation—a possible correlate of protection against *M. tuberculosis* infection. Clarifying the nature of the immune effector molecules secreted by microglia, and the means by which other CNS-specific cell types govern microglial activation or modulate their responses is critical if improved diagnostic and therapeutic strategies are to be attained. Therefore, this review evaluates the diverse roles microglia play in the neuro-immunity to *M. tuberculosis* infection of the CNS.

## Introduction

Microglia, from the literal Greek “*small glue*”, are one of three glial cell types found within the brain and spinal cord. As arguably the most prominent immune effector cells of the central nervous system (CNS), microglia simultaneously exhibit a potential for neurotoxicity. Found in numbers comparable to neurons, microglia comprise 0.5–16.6% of the total human CNS cell population, varying by anatomical site and with higher densities in white matter (Mittelbronn et al., 2001). Microglia are isolated within the cerebral parenchyma by the blood-brain barrier (BBB), thereby serving as the first line of defense against intra-cerebral infections such as *Mycobacterium tuberculosis* (*M. tuberculosis*), causative pathogen of tuberculosis (TB).

It is known that TB in the CNS (CNS-TB) generates host-orchestrated tissue destruction by infiltrating monocytes (Price et al., 2001; Lee et al., 2004). Although defined as the facultative phagocytic myeloid cells of the CNS, microglia are distinct from the macrophages located within the subarachnoid space, choroid plexus, meninges and perivascular spaces (Mittelbronn et al., 2001; Guillemin and Brew, 2004). Differential molecular expression, a unique “spiny” morphology, an experimentally-useful resilience to ionizing radiation and a unique blend of the phagocytic behavior of innate immune cells with the trophic nature of glia, define microglia as being a highly unique cell-type (Flaris et al., 1993; Ulvested et al., 1994; Giulian et al., 1995; Aarum et al., 2003; Guillemin and Brew, 2004; Balentova et al., 2015). This creates difficulty in relating the well-studied interactions between macrophage and *M. tuberculosis* of the periphery to the role microglia might play in CNS infections—an interaction which has, unfortunately, formed the basis of our understanding of microglia in CNS-TB infection to date.

Polarized views of beneficial and harmful results of active microglia have not clarified the perception of the diverse roles of microglia during CNS pathology (Glezer et al., 2007; Hanisch and Kettenmann, 2007; Sierra et al., 2013). On one hand, microglia are viewed positively as the initiators and sustainers of acute neuroinflammation where they are responsible for pathogen identification, the subsequent clearance of infection, insult repair and the restoration of cerebral homeostasis. Such trophic roles stand in stark contrast to their capacity for robust immune activation and their accountability for the consequent neuropathology of chronic inflammation. Therefore, this review aims to describe and critically evaluate the potential roles of microglia in the pathogenesis of *M. tuberculosis* infection of the CNS, a better understanding of which is critical for improved diagnostic and therapeutic technologies.

## Tuberculosis of the Central Nervous System

In 2013, an estimated 1.5 million people succumbed to TB, making it second only to HIV as the largest cause of infectious mortality. Global incidence remains high, estimated at 9 million cases (World Health Organisation (WHO), 2014). One in three people are thought to be latently infected, carrying a lifetime risk of developing active, transmissible disease. Extra-pulmonary TB accounts for 15–20% of all cases prior to the HIV pandemic (Mehta et al., 1991; Kulchavenya, 2014). Approximately 3–10% of all extra-pulmonary TB cases in developed countries exhibit CNS involvement (Rieder et al., 1990; Houston and Macallan, 2014), with far higher prevalence likely in developing countries bearing the brunt of the HIV pandemic (Berenguer et al., 1992; Leeds et al., 2012). Although CNS-TB represents just 1% of the global TB burden (Cherian and Thomas, 2011), it is the severest form of TB owing largely to its difficulty in diagnosis (Karstaedt et al., 1998; Marais et al., 2010), and high mortality and morbidity even after appropriate management (Afghani and Lieberman, 1994; Cheng et al., 2002)—with children and the immunosupressed being vulnerable, yet not presenting atypically (Dubé et al., 1992; Farinha et al., 2000; Nelson and Zunt, 2011). In African adults, approximately one in three cases of bacterial meningitis is attributable to *M. tuberculosis* infection, with fatality in almost two out of every three patients (Woldeamanuel and Girma, 2013).

The propensity for disseminated disease depends upon both bacterial and host-specific factors. Two retrospective studies found significant associations between strain-patterning and CNS infection (Arvanitakis et al., 1998; Click et al., 2012), whilst a Brazilian study using Restriction Fragment Length Polymorphism analysis concluded that risk factors for dissemination are more host-dependent (Gomes et al., 2013). A meta-analysis concluded that age, sex, and lifestyle habits, in addition to immunological factors (but not, interestingly HIV status), contributed towards the probability of extra-pulmonary TB (Webster and Shandera, 2014). This indicates that host factors are also critical to the pathogenesis of extra-pulmonary TB, as disease often results from either exaggerated or inefficient host-responses. Therefore, studying the role of host cells, such as microglia, is just as important as studying the pathobiology of the infectious agent.

Clinically, CNS-TB has been studied through the use of various imaging techniques, resulting in a hierarchical classification system. Primary classification is on the basis of the infection affecting the spinal cord or the cerebrum, then further sub-classified by the diffuse or localized nature of the infection, and finally, anatomically by the precise nidus of infection (Jinkins et al., 1995; Bernaerts et al., 2003). TB meningitis (TBM) is a diffuse infection of the leptomeninges, characteristically affecting the brain in a basal fashion (Thwaites and Hien, 2005). However, infection of the pachymeninges has also been described (Bernaerts et al., 2003). Direct infection of the brain parenchyma does occur, during which the *M. tuberculosis* bacilli breech the BBB. Localized infections of the parenchyma may result in a tuberculoma, an abscess, or focal cerebritis, whilst more diffuse parenchymal infections are, by definition, encephalitic. Such a diverse spectrum of cerebral infections has been explained through a unifying pathogenic theory, built largely upon the seminal studies by Rich et al. who posited that, following hematogenous deposition of bacilli into the parenchyma, the subsequent tuberculoma ruptures into the cerebral spinal fluid and adjacent brain structures become infected (Rich and Mccordock, 1933; Donald et al., 2005). However, many questions are left unanswered. For instance, what are the differential host mechanisms regulating BBB penetration by *M. tuberculosis*, and, if the parenchyma is required to be infected prior to cerebral dissemination, what immunological factors could potentially be mediating the disease. These questions lead one to consider the most renowned immunological effector cells of the CNS, the microglia, as the missing link in this paradigm.

## Microglia

### Origin and Maintenance

Historically, microglia were considered derivatives of invading pia, or malleable neuroectodermal elements (Rezaie and Male, 2002). Subsequent studies recognized their origin from mesoderm; borne from bone-marrow progenitors that seed the brain parenchyma (Hess et al., 2004). The resemblance of microglia to macrophages in surface antigen expression, as well as both phagocytic and cytotoxic effector functions hinted, particularly, at a myeloid origin. Although many experiments failed to provide definitive proof of such a myeloid heritage, mice lacking the myeloid-specific transcription factor *PU1.1* also lacked microglia (Beers et al., 2006). Later, primitive microglia were identified as erythromyeloid precursors arising from the yolk sack very early in embryogenesis (Alliot et al., 1999; Ginhoux et al., 2010; Schulz et al., 2012; Kierdorf et al., 2013). It was originally hypothesized that continual replenishment of the microglia population occurred into adulthood via peripheral recruitment of circulating monocytes, followed by subsequent differentiation steps. However, although murine monocytes have been shown to invade the CNS amidst insult (Andersson et al., 1992) and microglia demonstrate the potential to differentiate into either CNS-macrophage or dendritic cell profiles *in vitro* (Santambrogio et al., 2001), evidence for monocyte to microglial differentiation in the developed CNS is lacking (Ajami et al., 2007; Ginhoux et al., 2010). Thus, these studies suggest that microglia, upon successful CNS-seeding of their progenitors in early development, act as an independent, self-renewing population into adulthood (Ginhoux et al., 2010).

### Populations and Phenotypes

Microglial cells may be classified by location or functional morphology. Juxtavascular microglia, which contribute to the glia limitans by incorporating processes between those of astrocytes, are found adjacent to and migrate along penetrating cerebral arteries (Lassmann et al., 1991; Grossmann et al., 2002; Mathiisen et al., 2010). Microglia not in contact with the CNS microvasculature contribute to the parenchymal population. Perivascular antigen-presenting macrophages, ensheathed within the basal lamina and replenished by bone marrow progenitors (Hickey and Kimura, 1988; Hickey et al., 1992), are sometimes referred to as perivascular “microglia.” The opinion that true microglia are “macrophages of the CNS” is perhaps simplistic; advances in monocyte-macrophage immunology, such as the introduction of the M1-M2 paradigm or classical vs. alternative activation have been extrapolated from peripheral to central (i.e., microglial) immunological processes (Mittelbronn, 2014).

Nevertheless, microglial morphological plasticity reflects a specific yet stereotypical, graded spectrum of functional states. Ramified or “branched” microglia are a resting but highly active, baseline phenotype continually palpating the local microenvironment with cytoplasmic processes; searching for pathogens, signs of injury or homeostatic disturbances. Ramified microglia are well known for their potential to up-regulate the constitutive expression of both major histocompatibility complex (MHC) classes, amongst many other immune molecules (Leong and Ling, 1992; Ford et al., 1995; Olah et al., 2011). Activated microglia (upon encounter of injury or pathogen) typically display an amoeboid phenotype through cytoplasmic contraction. Such microglia are defined functionally by migration to the site of interest (Carbonell et al., 2005), proliferation (Giordana et al., 1994), discretional phagocytosis of self or non-self constituents (Magnus et al., 2001; Rogers and Lue, 2001; Shams et al., 2003), cytokine and chemokine expression (Hanisch, 2002), and induction of reactive oxygen species (ROS; Colton et al., 1996; Wang et al., 2004; Long et al., 2006). Such morphological diversity is further amplified by regional variations in molecular expression (de Haas et al., 2008), and evidence suggesting that microglial activation may simultaneously generate an immune-regulatory phenotype (Liao et al., 2012; Selenica et al., 2013).

## Microglia-*Mycobacterium tuberculosis* Interactions

### Microglia in Context: the Cellular and Biochemical Milieu

Microglia rely heavily on a complex system of *in vivo* signals from the surrounding cellular and biochemical milieu for both activation and modulation. Although microglia are the principal CNS cells infected by *M. tuberculosis* (Peterson et al., 1995a; Rock et al., 2005; Yang et al., 2007), other CNS-specific cells that display potential for *M. tuberculosis* infection include astrocytes and neurons (Rock et al., 2005; Randall et al., 2014). When human astrocytes and microglia were challenged with *M. tuberculosis in vitro*, Rock et al. observed a 15% and 76% bacilli uptake respectively (Rock et al., 2005). Teasing apart these complex cellular interactions, both amongst the different CNS cell-types and with the immune system, remains an outstanding step towards fully understanding the molecular pathogenesis of CNS-TB.

The most striking feature of microglial activation in general is the rapidity at which it occurs, suggesting a potential role of diminishing neuronal inhibitory signals in producing swift immune responses. Randall et al. (2014) were the first to observe that neurons can be infected by *M. tuberculosis* and, in response, contribute immunologically to the inflammatory state by secreting IL-1β, IL-6, and IL-10 (Randall et al., 2014). Although neither the extent to which this occurs *in vivo* during human CNS-TB pathogenesis nor the consequences of such infections on neuronal electrical or immunological signaling has been fully investigated, this introduces the possibility that altered neuron-microglia interactions (either in diminishing inhibitory signals, or increasing activation signals) as well as the direct participation of other CNS cell-types promote the pro-inflammatory milieu that drives chronic inflammation and culminates in subsequent host-pathology.

### *Mycobacterium tuberculosis* Recognition, Internalization and Microglial Activation

Microglia possess a unique repertoire of innate-immune and neuro-specific receptors, including pattern-recognition receptors (PRRs); the broad class of molecules used to identify pathogen-associated molecular patterns for self vs. non-self distinction. Although some of these receptors are of importance in macrophage recognition of *M. tuberculosis*, further experimental evidence is required to confirm their role in the microglial response to *M. tuberculosis* (Table 1).

**Table 1:** **Correlation between innate receptors on macrophages and microglia that have demonstrated importance in *M. tuberculosis* infections**.

Microglial PRR	Macrophage recognition of *M. tuberculosis*	Microglial recognition of *M. tuberculosis*	References
TLR2	+	Unknown	Drennan et al. (2004) and Kielian et al. (2005)
TLR4	+	Unknown	Abel et al. (2002)
TLR9	+	Unknown	Bafica et al. (2005)
CD14	−	+	Peterson et al. (1995a), Means et al. (1999), and Shams et al. (2003)
CR3 (CD11b/CD18)	+	Unknown	Melo et al. (2000)

Comparing the experimentally determined importance of innate immune receptors in M. tuberculosis infection of macrophages and microglia. “+” indicates confirmed importance, “−” indicates no importance determined whilst “unknown” indicates a lack of experimental evidence altogether

Internalization of *M. tuberculosis* bacilli by human microglia is dependent on CD14 – a monocyte differentiation antigen which binds to lipopolysaccharide (LPS) with Toll-like receptor 4 (TLR4; Wright et al., 1990). Peterson et al. (1995a) observed a 64% and 62% reduction in non-opsonized tubercle bacilli uptake in the presence of anti-CD14 monoclonal antibodies and soluble CD14 ligand, respectively (Peterson et al., 1995a). On the other hand, Shams et al. (2003) found that CD14 does not mediate entry of *M. tuberculosis* into human peripheral blood mononuclear cells (Shams et al., 2003), while others observed a CD14-dependant and regulated internalization of *M. bovis* (Khanna et al., 1996; Sendide et al., 2005). Dectin-1 and TLR2 have been recognized as key mediators of macrophage activation by *M. tuberculosis* (Yadav and Schorey, 2006). Yang et al. used combinations of well-characterized TLR2 antigen, dectin-1 antagonists and TLR2-deficient mice to show that *M. tuberculosis* bacilli recognition by microglia occurs via an as yet unidentified pathogen recognition mechanism involving identification of a heat-stable *M. tuberculosis* bacilli antigen (Yang et al., 2007). Such recognition could possibly be orchestrated by other PRR’s or perhaps activate through alteration of microglial-specific ion channels (Kettenmann et al., 1990; Prinz et al., 1999), which have been shown to be modulated by both cytokine signals and pathogen associated molecular patterns such as LPS (Nörenberg et al., 1994). Interestingly, Lambert et al. has shown the induction of dendritic cell-specific intercellular adhesion molecule grabbing nonintegrin (DC-SIGN) in human microglia when treated with GM-CSF, IL-4, and LPS (Lambert et al., 2008). DC-SIGN is a known PRR expressed by DC as part of the innate immunity for the recognition of *M. tuberculosis* (Tailleux et al., 2003), therefore one cannot exclude the potential recognition of *M. tuberculosis* by the induced microglia using DC-SIGN.

Whilst most pathogens attempt to avoid host immunity, it is generally accepted that tubercle bacilli actively seek internalization by host macrophages in which they have developed strategies to survive. Of all the parenchymal CNS cell types, microglia could, theoretically, demonstrate preferential infection by *M. tuberculosis* due to their similarity with monocytes, as evidenced by their tendency to associate with more bacilli per cell than astrocytes (Rock et al., 2005). Microglia internalize virulent *M. tuberculosis* more rapidly and efficiently than less virulent strains. Upon internalization, tubercle bacilli are found in sparse, but densely packed “vacuoles” (Curto et al., 2004). A number of studies report *M. tuberculosis* bacilli retaining reproductive potential within infected microglia; providing a cerebral niche for persistence and a possible mechanism for subsequent reactivation should a state of immune-suppression be acquired (Peterson et al., 1995b; Curto et al., 2004; Cannas et al., 2011).

To illustrate the complexity of immune-modulatory signals, microglia have diminished phagocytic capacity when treated with anti-CD14 antibodies, opiate antagonists and pertussis toxin; indicating a G-protein dependent mechanism (Peterson et al., 1995b). Although opiate abuse has been associated with CNS-TB development, Peterson et al. (1995b) observed an enhanced phagocytic capacity of primary fetal microglia when pre-exposed to a morphine concentration of 10^−^M—reporting a higher proportion of phagocytically active microglia and greater *M. tuberculosis* burdens (Peterson et al., 1995b). Mu receptors have also been implicated in the control of microglial chemotaxis, suggesting that morphine’s anti-inflammatory action is due to a reduction in microgliosis rather than diminished microglial activity (Chao et al., 1997).

Microglial activation by *M. tuberculosis* has been largely studied through cytokine and chemokine as opposed to morphological or transcriptional responses. Messenger RNA and protein expression studies by Qin et al. (2015) suggest that a classically activated, M1 phenotype is induced and an M2-like phenotype reduced in microglia following exposure to *Mycobacterium*-challenged macrophage culture medium (Qin et al., 2015). Although such studies need to be verified in a human model, several important observations can be made. Firstly, this illustrates that microglia, even when not directly infected, can be activated and respond immunologically to infections by tubercle bacilli. Secondly, macrophage infection by *M. tuberculosis* may not only incite a pro-inflammatory microglial phenotype, but their persistent infection may prevent either the conversion or reversion of microglia to a more M2-like phenotype. This has further potential applications to the underlying pathophysiology of CNS-TB as macrophage infection could: precede CNS-parenchymal infection; contribute towards a breakdown of the BBB for peripheral immune recruitment or enhance bacilli breech; contribute towards either the activation or persistence of a pro-inflammatory milieu through direct communication with microglia.

Furthermore, patterns of microglial activation differ not only between organisms, but also between virulent and avirulent strains of mycobacteria—revealing the high degree of specificity with which microglia respond to pathogens (Curto et al., 2004; Cannas et al., 2011). Curto et al. challenged the “all-or-nothing” dogma of microglial activation by observing a stronger inhibition of both IL-1 and IL-10 in *M. tuberculosis*-microglial infections with more virulent strains (Curto et al., 2004). This finding suggests that *M. tuberculosis* infection initiates a rigorous transcription profile that enhances the expression of certain molecules whilst simultaneously suppressing the expression of others. Therefore, microglial effector mechanisms are tightly regulated, pathogen-specific responses that appear to also be virulence-specific, and such effector profiles, or a dysregulation in certain profiles, may yet be correlated with either a propensity to neuropathology, or a heightened resistance to it.

### Cytokine Effector Responses Orchestrated by Microglia

**Figure 1:**
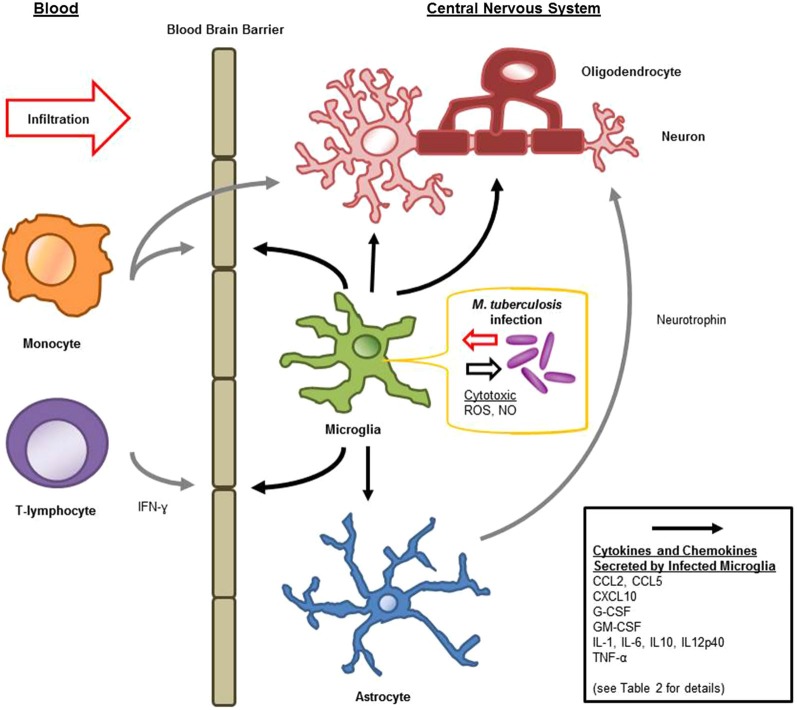
**Schematic diagram of microglial immune responses and interactions during *M. tuberculosis* infection in the central nervous system (CNS)**.

Microglia are known to be capable of secreting a wide range of cytokines and chemokines (Figure 1). The cytokine levels in TBM are distinct from meningitis caused by other microbes (Mastroianni et al., 1998). Studies in cerebrospinal fluid of patients with CNS-TB indicate significantly elevated concentrations of sTNFR-75, sTNFR-55, IFN-γ, and IL-10, and persistently elevated levels of TNF-α—even following therapeutic interventions (Mastroianni et al., 1997). Other immunological molecules, confirmed experimentally, to be secreted by microglia following *M. tuberculosis* stimulation include: IL-1α, IL-1β, IL-6; IL-10, IL-12p40, TNF-α, G-CSF, GM-CSF, CCL2, CCL5, and CXCL10 (Curto et al., 2004; Rock et al., 2005; Yang et al., 2007; Cannas et al., 2011; Table 2). In comparison, Rock et al. found *M. tuberculosis*-challenged astrocytes to have a much narrower cytokine-chemokine response—detecting modest levels of CXCL10 only (Rock et al., 2005).

**Table 2:** **Experimentally confirmed cytokine and chemokine expression by microglia during *M. tuberculosis* infection summarizing the experimental evidence for microglial cytokine secretion in response to *M. tuberculosis* stimulation**.

Molecule	Microglia line	*M. tuberculosis* strains	Experimental model	Reference
CCL2/ MCP1	Human Fetal	H37Rv	Human	Rock et al. (2005)
CCL5/RANTES	Human Fetal	H37Rv	Human	Rock et al. (2005)
CXCl10	Human Fetal	H37Rv	Human	Rock et al. (2005)
G-CSF	Murine BV-2	H37Rv	Murine	Cannas et al. (2011)
GM-CSF	Murine BV-2	H37Rv	Murine	Cannas et al. (2011)
IL-1	Human Fetal	N.C 0741708	Human	Curto et al. (2004)
IL-1α	BV-2	H37Rv	Murine	Cannas et al. (2011)
IL-1β	BV-2; Human Fetal	H37Rv	Murine, Human	Rock et al. (2005) and Cannas et al. (2011)
IL-10	Human Fetal	N.C 0741708	Human	Curto et al. (2004)
IL-12p40	BV-2	H37Rv	Murine	Yang et al. (2007)
IL-6	BV-2; Human Fetal	H37Rv	Murine, Human	Rock et al. (2005) and Yang et al. (2007)
TNF-α	BV-2; Human Fetal	H37Rv; N.C 0741708;H37Rv	Murine, Human	Curto et al. (2004), Yang et al. (2007) and Cannas et al. (2011)

TNF, a pro-inflammatory cytokine, demonstrates paradoxically destructive and protective roles in CNS and peripheral *M. tuberculosis* infection, owing to transmembrane or soluble forms binding to either of two receptors. Microglia encountering *M. tuberculosis*, generate an initial burst of TNF-α, followed by a sustained decline thereafter (Curto et al., 2004). TNF-α acts on other microglia, possibly in an autocrine fashion, to induce NADPH production of H_2_O_2_, and thus drive microglial proliferation—an attempt either to promote neuroinflammation or limit its sequelae (Mander et al., 2006). TNF-α has been shown to have both protective (Nawashiro et al., 1997) and harmful (Barone et al., 1997) effects in cerebral ischemia, and alone induces necrotic changes in cerebral endothelial cells. After which, microglia could contribute towards forming a secondary BBB (Claudio et al., 1994). Multi-nucleated giant cell formation following *M. bovis* is initiated by TNF-α in swine microglia (Peterson et al., 1996), making TNF-α a critical contributor to the formation of tuberculomas and the subsequent seclusion of mycobacteria. TNF-α induces expression of chemokines within the CNS, including intercellular adhesion molecule (ICAM-1), important in leukocyte recruitment to the brain during bacterial infection (Engelhardt et al., 1994). Although TNF-α appears to be the most potent ICAM-1 inducer within the CNS, it acts in concert with other molecules in the recruitment of leukocytes into the nervous system (Claudio et al., 1994; Shrikant et al., 1994; Glabinski et al., 2003). Interestingly, neurotoxicity has been attributed to the synergism of TNF-α with IL-1β on NO generation by astrocytes (Chao et al., 1995). Moreover, the importance of TNF has been further supported by the neutralization study, leading to *M. tuberculosis* dissemination causing severe CNS-TB (Seong et al., 2007; Lynch and Farrell, 2010).

IL-1α and IL-1β, members of the pyrogenic IL-1 family of cytokines, are both believed to act through IL-1RI with an accessory protein, and compete for binding with IL-1Rα. Although secreted, a strong inhibition of IL-1α expression was observed when microglia were infected with more virulent mycobacteria (Curto et al., 2004; Cannas et al., 2011). Given the importance of IL-1α in a pulmonary granulomatous response (Kasahara et al., 1988), both the initial parenchymal cell type and the virulence of the mycobacteria infection may dictate the IL-1α levels within the CNS, and thus define both the form and course of the infection. IL-1β has also been shown to be secreted by both neurons and microglia upon encountering *M. tuberculosis* (Cannas et al., 2011; Randall et al., 2014), and is known to induce microglial proliferation via the same mechanism as TNF-α (Mander et al., 2006). Recombinant IL-1β injected into rat brain induces both astrocyte proliferation and stimulates blood vessel growth (Giulian et al., 1988), and has been shown to initiate transcription of type II NOS (Liu et al., 1996).

Astrocytes play an important role in complementing and counteracting the adverse effects of IL-1α and IL-1β by secreting G-CSF and GM-CSF in response (Tweardy et al., 1990). Murine microglia have also been shown to secrete G-CSF and GM-CSF when infected with *M. tuberculosis*, which, unlike IL-1 or IL-2, drives microglial-specific proliferation via JAK/STAT pathways (Lee et al., 1994; Liva et al., 1999), amoeboid differentiation, and stimulates debris clearance (Giulian and Ingeman, 1988; Cannas et al., 2011). GM-CSF may facilitate bacilli containment by augmenting the neutrophilic phagocytosis of bacilli (Fleischmann et al., 1986) and enhancing the bactericidal mechanisms of macrophages (Blanchard et al., 1991)—but whether G-CSF contributes to collateral neurotoxic damage through infiltrating leukocyte activation remains to be investigated. Some systemic symptoms of CNS-TB may be due to GM-CSF negatively affecting food intake and positively affecting energy expenditure (Reed et al., 2005). G-CSF, like GM-CSF, is mainly known for its hematopoietic effects. Recently, however, it has been recognized that G-CSF plays important roles in the induction of immune tolerance, including redirection of T-lymphocytes to a Th2 phenotype (Pan et al., 1995; Sloand et al., 2000), and of specific importance in *M. tuberculosis* immunity, a decline in IFN-γ secretion (Sloand et al., 2000). G-CSF and GM-CSF not only augment phagocyte cell survival, but also highly neurotrophic factors: decreasing cortical ischemic damage, improving neuronal survivability and contributing towards neuronal regeneration (Kim et al., 2004; Schneider et al., 2005). Thus, microglia do not only orchestrate and lead the host immune response (and through which, may participate in neurotoxicity), but may have additional roles in neuronal protection and recovery.

Microglia secrete IL-6 in direct response to *M. tuberculosis* (Yang et al., 2007) and, through TNF-α, promote its additional expression by astrocytes (Sawada et al., 1992), and serves to dampen the TNF-inducible expression of VCAM-1 within the CNS (Oh et al., 1998). IL-6, well-known for its primary importance in B-lymphocyte differentiation (Burdin et al., 1995), has secondary, neuron-specific effects. IL-6 induces neurotrophin secretion from astrocytes in a region-specific pattern: (März et al., 1999) this not only disrupts the IFN-γ-induced expression of MHC class II by microglia (Neumann et al., 1998), but has been shown to increase the survival of dissociated neurons (Thier et al., 1999). Monocytes cultured from Chinese individuals with rs1800796GG polymorphisms produced less IL-6, which also granted these individuals a reduced risk of pulmonary TB (Zhang et al., 2012). This correlates with observations that *M. tuberculosis* bacilli maximize IL-6 production from macrophages to antagonize IFN-γ-induced autophagy, increasing the longevity of their macrophage host and thereby extending intracellular persistence (Dutta et al., 2012). Although not yet investigated, IL-6 may thus be used by the tubercle bacilli to extend the lifespan of their CNS-specific host-cells, including (but not limited to) microglia and neurons (Randall et al., 2014).

Neurons infected with *M. tuberculosis* act as a source of IL-10 which is generally accepted as an anti-inflammatory cytokine. In TB specifically, its principal role is considered to be the regulation of Th1 responses and thus, opposing IFN-γ production (Jamil et al., 2007). In the CNS, IL-10 diminishes MHC class II receptor expression on microglia but not astrocytes, and reduces the proliferative response induced by glial interactions with effector T-lymphocytes (Frei et al., 1994). IL-10 may also silence cytokine production by infiltrating monocytes (de Waal Malefyt et al., 1991). IL-10 represents an important mechanism by which the body protects itself from CNS-autoimmunity through Th1 attenuation (Bettelli et al., 1998; Fillatreau et al., 2002). Furthermore, Curto et al. observed inhibition of IL-10 expression by microglia in more virulent *M. tuberculosis* infection, uncovering one possible mechanism driving a pro-inflammatory response, and potentially the basis of pathogenicity between mycobacteria within the CNS (Curto et al., 2004). Considering IL-10 alone suggests possible contributory mechanisms in TB neuropathology: loss of neuron-to-effector inhibition, which results in spurious immune activation; a relative resistance of microglia in some individuals, be the cause acquired or inherited, resulting in autonomous immune activation; or a relatively pro-inflammatory milieu (to which multiple cells contributed) as ultimately generating host-mediated tissue damage.

### Potential Neurotoxicity

The inflammatory damage found in the CNS amidst infections, ranging from HIV (Garden, 2002) to bacterial meningitis (Gerber and Nau, 2010) has, of all the CNS-specific cells-types, been attributed largely to microglia. However, microglia are not the only cell type to produce increased MMPs (matrix metalloproteinases) in response to TB, and neither do microglia act in isolation. It is, in fact, the contribution of other cell types towards a pro-inflammatory milieu that appears to drive the secretion of destructive compounds. For example, *M. tuberculosis*-activated monocytes release factors into the local microenvironment that rapidly stimulate microglia to produce MMP-1 and MMP-3, well known to induce tissue damage through degradation of various matrix-associated proteins (Green et al., 2013). The dependency on monocyte-priming of microglia was reproduced in other studies, where it was reported that significantly greater MMP-1, MMP-3, and MMP-9 synthesis is observed in microglia co-cultured in *M. tuberculosis*-infected monocyte culture medium as opposed to those microglia exposed to *M. tuberculosis* bacilli alone (Green et al., 2010). Astrocytes are an additional source of MMPs within the CNS, and have been shown to secrete significantly more MMP-9 in a monocyte-dependent fashion as do microglia (Harris et al., 2007). Such data suggests that it is miscommunication or dysregulation between many cell types, performing exaggerated but physiological activities, that result in CNS pathology, rather than individual cellular populations (like microglia) inducing pathology autonomously.

Although it has not been studied directly in the context of *M. tuberculosis*, microglia are known to demonstrate cytotoxic behavior towards oligodendrocytes—a possible component of demyelinating tuberculous diseases. One such rarer form of CNS-TB, Tuberculous (allergic) encephalopathy, usually occurs in vulnerable populations with a preceding or concurrent tuberculous infection (Bernaerts et al., 2003). The broad pathognomonic features of this complication are diffuse white matter destruction, occurring with or without clinical meningism in an individual exposed to TB, and has been classically attributed to a delayed hypersensitivity reaction towards tuberculoprotein (Udani and Dastur, 1970; Dastur, 1986). Activated microglia are capable of lysing oligodendrocytes via a NO-dependent mechanism requiring membrane-bound TNF-α (Merrill et al., 1993). Alternatively, a more novel mechanism whereby microglia destroy oligodendrocytes involves a local spike in extracellular glutamate and excitotoxic cellular death (Domercq et al., 2007). Regardless of the precise mechanisms, such microglial-oligodendrocyte interactions are well worth further investigation and may very well broaden the understanding of CNS-TB.

Also, many of the signaling molecules used by microglia are also potential sources of collateral neurotoxicity. Central to microglial pro-inflammatory activation pathways is secretory phospholipase A_2_ (sPLA_2_; Yang et al., 2009), a compound shown to be a culprit of toxicity in neurons (Kolko et al., 1996, 1999) and potential contributor to neural damage amidst CNS-TB. Studies in lungs have demonstrated the regulatory role of sPLA_2_ in inflammation through the induction of cytokine production and cellular recruitment (Granata et al., 2005, 2006). Yang et al. has shown in the CNS that sPLA_2_ is essential for *M. tuberculosis*-dependent ROS (in particular, H_2_O_2_ and superoxide) generation in microglia via increased NADPH oxidase activity, which further initiates MAPK signaling of the pro-inflammatory response (Yang et al., 2007). Tumor necrosis factor alpha (TNF-α) and IL-6 secretion are positively regulated by ERK1/2 and p38, but p38 alone negatively regulates IL-12p40 generation (Yang et al., 2007). IL-12 is critically important for the protective granulomatous, antigen-specific Th1 and CD8^+^ T-lymphocyte responses to *M. tuberculosis* infection (Hölscher et al., 2001). Hence, many of the pro-inflammatory programs initiated by microglia require potentially cytotoxic compounds.

Another potential source of cytotoxicity in CNS-TB is associated with the treatment of adjunctive corticosteroids. Its success is often attributed solely to the modulation of microglial pro-inflammatory cytokine activity, and thus used as evidence for the destructive nature of microglia. However, a double-blinded randomized control trial found that adjunctive corticosteroid use in patients with TB meningitis had improved mortality, but not morbidity (Thwaites et al., 2004). Furthermore, there was no difference in cytokine levels within the CNS of CNS-TB patients treated with corticosteroids (Claudio et al., 1994) and those who remained untreated (Mastroianni et al., 1997).

IL-1β and TNF-α are factors secreted from microglia that drive the production of MMP-2 and MMP-9 from astrocytes; their production reduced through glucocorticoids (Gottschall and Deb, 1996). Experiments involving dexamethasone demonstrate two main mechanisms by which the effects of corticosteroids in CNS-TB may be explained. Firstly, production of IL-1β, IL-6, and TNF-α by *M. tuberculosis*-stimulated microglia is significantly reduced (Rock et al., 2005). Secondly, dexamethasone reduces MMP-1 and MMP-3 production within the CNS (which could alternatively be explained by the reduction of its TNF-α and IL-1β, as these promote MMP secretion) (Green et al., 2010). However, microglia are not the only sources of these cytokines in the CNS, and thus to either fully achieve anti-inflammatory effects *in vivo* or to prove the beneficial effects of adjunct steroid use relating only to microglia attenuation, it is necessary to look at these cells in a much broader context. In other words, microglia could still respond to cytokines from additional, upstream sources, even if their own autocrine or paracrine responses have been suppressed.

### Blood-Brain Barrier Permeability and Immune Recruitment

Cytokines are generally considered inducers of BBB permeability for the influx of peripheral immune constituents (Figure 1). IL-6 and TNF-α increase cerebral endothelial cell permeability both *in vitro* and *in vivo* (Bamforth et al., 1996; Duchini et al., 1996). IL-1β interference of the BBB is associated with inter-endothelial pores, as well as leukocyte recruitment and hemorrhage (Claudio et al., 1994). Although these secreted products of microglia compromise the integrity of the BBB, this may facilitate additional activation signals: such as ATP by means of purinergic receptors, or complement through complement receptors (Lynch et al., 2004; Davalos et al., 2005). Microglia have been shown to internalize many extravasated proteins during BBB compromise (Claudio et al., 1994). Furthermore, as a component of the glia limitans capable of migration and immune activation, microglia not immediately responding to the intra-cerebral threats, may play a role in protecting the CNS when it is at its most vulnerable; compensating for the altered BBB permeability (Claudio et al., 1994).

Chemokines produced by *M. tuberculosis*-challenged microglia include CCL2, CCL5, and CXCL10 (Rock et al., 2005). CCL2 (also known as MCP-1) is essential for the cellular response in *M. tuberculosis* infection, recruiting leukocytes (in particular, monocytes, and T-lymphocytes) to the sites of infection or injury (Babcock et al., 2003; Hasan et al., 2005). Similarly, CCL5 has shown particular importance in recruiting T-lymphocytes in pulmonary granulomas (Berenguer et al., 1992; Vesosky et al., 2010), and CXCL10 (from microglia and *M. tuberculosis*-challenged astrocytes) is likely important in helper T-lymphocyte trafficking (Fife et al., 2001; Rock et al., 2005). Along with this importance in cellular recruitment into the CNS, CCL2 has shown additional inflammation-modulating and protective activities. CCL2 deficient mice have more pronounced pro-inflammatory responses from astrocytes (Semple et al., 2010). Both CCL2 and CCL5 increase neuronal resilience to neurotoxicity in various experimental settings (Bruno et al., 2000; Madrigal et al., 2009). Given that microglia express PRR’s, are preferentially infected by *M. tuberculosis* and secrete an array of immunologically-relevant molecules, it is likely that they are critical regulators of chemokine receptors within the CNS, hence regulating both the trafficking and the state of activation of peripheral immune components.

Throughout the course of mycobacterial infections of the CNS, infiltrating cell populations change, with significant recruitment of innate CD11b^+^ cells, CD11c^+^ cells, and CD4^+^ T-lymphocytes (Lee et al., 2009). Of these cell types recruited to the infected CNS, T-lymphocytes and monocytes have the best-characterized roles in TB. Within macrophages, *M. tuberculosis* evade intracellular killing by means of multiple mechanisms, including phago-lysosome exploitation and disruption of CD4+ T-lymphocyte interactions by a reduction of MHC Class II expression (Noss et al., 2001; Vergne et al., 2005). Although this may hold true for infiltrating monocytes, who could be the actual agents of neurotoxicity, the uniqueness of microglia makes it difficult to assume similar escape mechanisms within or MHC class II evasion in glia, and highlights the importance of gaining an experimental, rather than a purely hypothetical understanding, of such processes.

Microglia form an important mediator between innate and adaptive immune responses within the CNS. Microglial stimulation by *M. tuberculosis* induces the rapid expression of the co-stimulatory molecule CD137 for the activation of infiltrating T-lymphocytes (Curto et al., 2004). Numerous studies have highlighted the importance of a T-cell response against *M. tuberculosis*, in particular the importance of a robust Th1 response (Salgame, 2005). For instance, Lienhardt et al. found that African TB patients had not only reduced proxies of a Th1 response, but exhibited an inferior capacity to suppress a Th2 response (Lienhardt et al., 2002). Taking this into account, it is of utmost importance to appreciate microglia as a source of IL-12 amidst *M. tuberculosis* infection, which may play a role in polarizing local Th1 responses (Yang et al., 2007). Furthermore, IFN-γ treated microglia rapidly express MHC class II, through which they present antigen to helper T-lymphocytes (Frei et al., 1987; Steiniger and Van Der Meide, 1988) (as previously discussed, microglia may play roles in the regression thereof, too). IFN-γ, interestingly, also provides a mechanism to keep microglia in check by inducing apoptotic pathways (Spanaus et al., 1998; Badie et al., 2000). Although microglia are weaker antigen presenting cells compared to macrophages, they are perfectly adapted to the delicate CNS: stimulating the Th1 differentiation of T-lymphocytes without inducing their proliferation (Carson et al., 1999).

A number of meta-analyses have confirmed the efficacy of BCG against CNS-TB (Rodrigues et al., 1993; Colditz et al., 1994; Trunz et al., 2006). Whether this protective effect is due to preventing the bacilli from reaching the CNS, or through CNS-specific immune augmentation, remains to be uncovered. In a murine study of intracerebral BCG infection, Lee et al. report that microglia are the eminent TNF-α producers, with additional sources including infiltrating CD4^+^ T-lymphocytes (Lee et al., 2009). Given that microglia have the capacity to differentiate Foxp3^+^CD4^+^ T-regulatory lymphocytes (Ebner et al., 2013), and that unique IFN-γ^+^IL-17^+^ T-lymphocytes have been identified with protective immunity characterized by the Foxp3^+^CD4^+^ T-regulatory phenotype (Colditz et al., 1994), it is not unreasonable to assume that microglia may be involved in mediating the CNS efficacy of BCG against *M. tuberculosis*.

## Conclusion

To improve patient outcomes following CNS infections by *M. tuberculosis*, more research needs to be conducted on the mechanism of *M. tuberculosis* identification and internalization within the CNS, mechanisms of persistence within microglia, the nature of each cytokine or chemokine secreted by microglia, and the means by which other CNS specific cells responding to or infected by *M. tuberculosis* govern microglial activation and modulate their responses. The neurological environment is unique in that inflammatory signals, which may appear to be damaging in the periphery, may in fact be beneficial in the CNS by governing neuronal survival, regeneration and differentiation.

In conclusion, microglia are the understudied arbiters of initiating, maintaining within acceptable limits, and attenuating the immune responses to CNS-TB, and may even be critical in mediating the protection or recovery from such responses. During *M. tuberculosis* infection, microglia are essentially the conductors of a tightly regulated immune symphony, and may well be a missing link towards fully understanding the molecular pathogenesis of CNS-TB.

## Conflict of Interest Statement

The authors declare that the research was conducted in the absence of any commercial or financial relationships that could be construed as a potential conflict of interest.

## Abbreviations

BCG: Bacillus Calmette-Guérin
CCL: chemokine C-C motif ligand
CD: cluster of differentiation
CNS: central nervous system
CNS-TB: tuberculosis of the central nervous system
CSF: cerebrospinal fluid
CXCL: C-X-C motif chemokine
DC-SIGN: dendritic cell-specific intercellular adhesion molecule grabbing nonintegrin
ERK: extracellular-signal-regulated kinases
G-CSF: granulocyte colony stimulating factor
GM-CSF: granulocyte macrophage stimulating factor
LPS: lipopolysaccharide
MAPK: mitogen-activated protein kinase
IL: interleukin
MCP: monocyte chemotactic protein
MHC: major histocompatibility complex
MMP: matrix metallometalprotease
*M. tuberculosis*: *Mycobacterium tuberculosis*
NADPH: nicotinamide adenine dinucleotide phosphate
NOS: Nitric Oxide Synthase
PRR: pattern recognition receptor
ROS: reactive oxygen species
sPLA_2_: secretory phospholipase A_2_
TB: Tuberculosis
TLR: toll like receptor
TNF: Tumor necrosis factor
ICAM: intracellular adhesion molecule.
